# Why study sleep in flatworms?

**DOI:** 10.1007/s00360-023-01480-x

**Published:** 2023-03-11

**Authors:** Shauni E. T. Omond, John A. Lesku

**Affiliations:** School of Agriculture, Biomedicine & Environment, La Trobe University, Melbourne, Australia

**Keywords:** *Girardia*, Neurogenesis, Neurotransmitter, Regeneration, *Schmidtea mediterranea*, Sleep function

## Abstract

The behaviors that characterize sleep have been observed across a broad range of different species. While much attention has been placed on vertebrates (mostly mammals and birds), the grand diversity of invertebrates has gone largely unexplored. Here, we introduce the intrigue and special value in the study of sleeping platyhelminth flatworms. Flatworms are closely related to annelids and mollusks, and yet are comparatively simple. They lack a circulatory system, respiratory system, endocrine glands, a coelom, and an anus. They retain a central and peripheral nervous system, various sensory systems, and an ability to learn. Flatworms sleep, like other animals, a state which is regulated by prior sleep/wake history and by the neurotransmitter GABA. Furthermore, they possess a remarkable ability to regenerate from a mere fragment of the original animal. The regenerative capabilities of flatworms make them a unique bilaterally symmetric animal to study a link between sleep and neurodevelopment. Lastly, the recent applications of tools for probing the flatworm genome, metabolism, and brain activity make their entrance into the field of sleep research all the more timely.

## Introduction

In sleep research, and indeed much of neuroscience, a great deal is known from a great few species (Manger et al. 2008). That is, our collective insight into sleep and its sub-states, neural circuitry, circadian and homeostatic regulation, cellular and molecular expression, and processes and purpose, all derive (overwhelmingly) from the study of humans and laboratory-bred rodent strains. This bias offers depth of understanding at the expense of breadth (Blumberg et al. 2020; Eban-Rothschild 2022; Lesku and Rattenborg 2022). Lauded efforts have been made to diversify our study of sleep, through investigations into other model systems, such as the zebrafish (*Danio rerio*), fruit fly (*Drosophila melanogaster*), and nematode (*Caenorhabditis elegans*). Nonetheless, much work remains (Siegel 2008). Although the study of sleep in 8.7 million animal species would be impractical, we can be smart with sampling selections. Animals which exhibit unique life histories and extreme lifestyles might be most telling (Lesku and Rattenborg 2022). So too are species perched on key evolutionary branches, and those capable of fascinating physiological feats.

Aside from many vertebrates, for which measures of brain activity are the chief means to measure to sleep (Ungurean et al. 2020; Rattenborg et al. 2022), sleep is often characterized by behavior alone in other groups (e.g., Ajayi et al. 2022). Here, sleep is recognized by reduced responsiveness during periods of restfulness. With sufficiently strong stimulation, restfulness gives way to wakefulness, and deprivation of the sleep state leads to a rebound. Using this behavioral assay, it has been possible to demonstrate the presence of sleep in six animal phyla (Rattenborg and Ungurean 2023), including brainless cnidarians, represented by the upside-down jellyfish (*Cassiopea* spp.) and *Hydra* (Nath et al. 2017; Kanaya et al. 2020).

### What are flatworms?

**Fig. 1:**
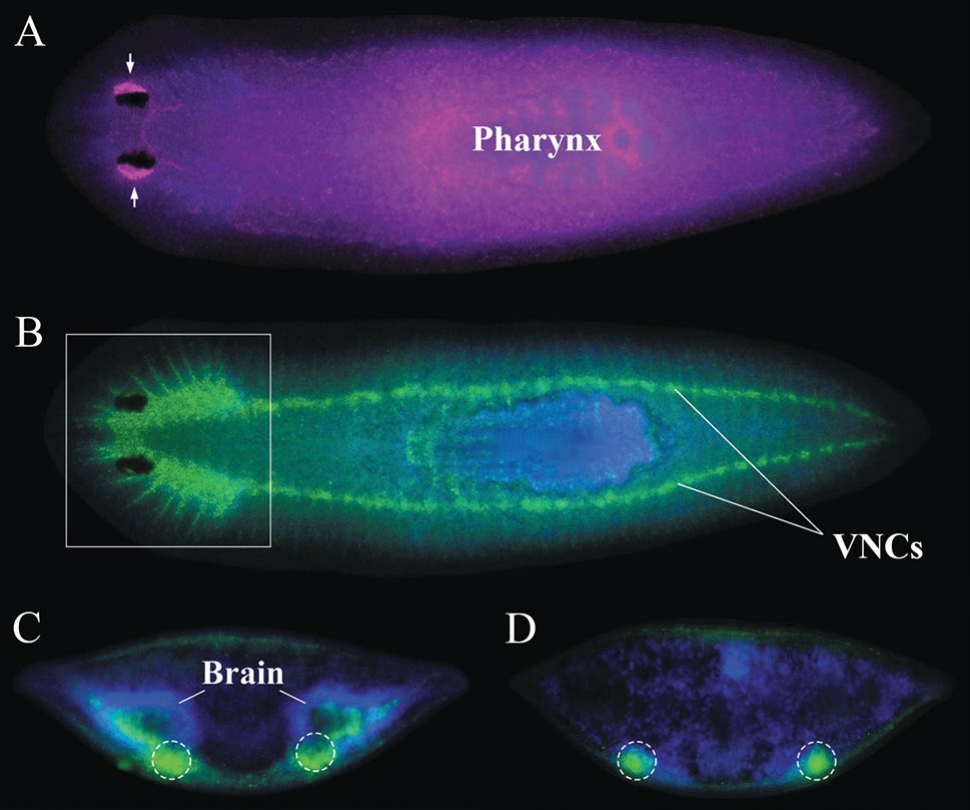
**A** Dorsal view of a magenta stained flatworm (*Dugesia japonica*) using a muscle-specific probe highlighting the pharynx and pharyngeal opening on the ventral surface; photoreceptor cells and their axons are visible in the anterior region (*arrows*). **B** Antibody staining of the same animal as in **A** emphasizing the central and peripheral nervous systems; specifically, the axonal networks of the brain (*box*) and pair of ventral nerve cords (*VNCs*) running down the length of the body. Transverse view of **C** the head at a level that includes the bilobed brain and **D** the VNCs. The brain consists of nerve cells (*blue*) and a core of axons (*green*) located nearer the dorsal surface than the VNCs (*dotted circles*). Reprinted with permission from Umesono and Agata (2009) (color figure online)

Flatworms are small (< 10 mm length) free-living, mostly aquatic invertebrates of the phylum Platyhelminthes. They are bilaterally symmetric, in contrast to the radial symmetry of cnidarians and ctenophores, and the asymmetry of sponges. Near the midpoint of the body is the pharynx through which the animal both eats and excretes waste (Fig. 1A). They have a bilobed brain located toward the anterior end of the animal, and two ventral nerve cords that run much of the length of their body (Fig. 1B–D). Flatworms have diverse sensory systems, including sensitivities to light (Paskin et al. 2014) and chemical gradients (Miyamoto and Shimozawa 1985), and electric (Brown et al. 1968) and magnetic (Brown and Chow 1975) fields. They can also learn via classical conditioning (Lee 1963) and have shown long-term memory (Best and Rubinstein 1962; Shomrat and Levin 2013; reviewed in Deochand et al. 2018).

Whether the daily timing of flatworm behavior is shaped by a circadian clock is unknown, but converging lines of evidence are suggestive of a clock in flatworms. A molecular time-keeper could come from the expression of the gene *Smed-Tim* in planarians, a homolog of the mammalian clock gene *Tim* (Tsoumtsa et al. 2017). More conspicuously, flatworms are less active during the day—a diel rhythm that persists under constant dark conditions—and they appear to anticipate photoperiodic change (Omond et al. 2017; Hinrichsen et al. 2019). Furthermore, the production of melatonin, serotonin, and arylalkylamine N-acetyltransferase vary over a 24-h day under both 12:12 light:dark and constant dark regimes (Itoh et al. 1999; Itoh and Igarashi 2000). That said, future study is needed to determine whether flatworms have a circadian clock characterized by entrainment and temperature compensation.

**Fig. 2:**
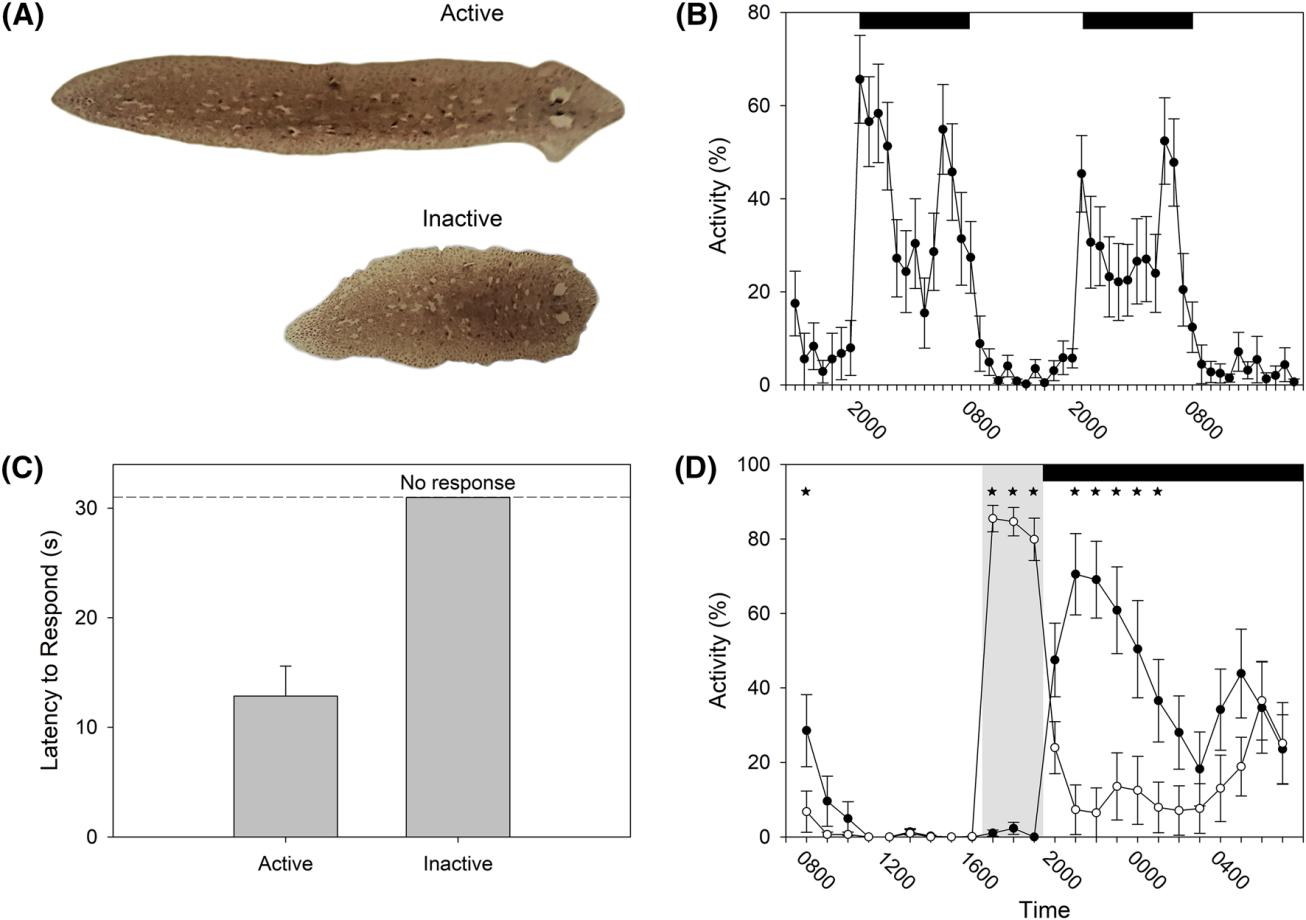
**A** Photographs of a flatworm (*Girardia tigrina*) in its two behavioral states: extended (10 mm long) gliding along the substrate, and a contracted animal that is restful. Two eyespots are visible in the anterior end (*right*). **B** Activity patterns reveal flatworms to be nocturnal under a 12:12 light:dark photoperiod; the black horizontal bars along the top of the plot denote night-time. **C** Flatworms respond quickly to an overhead dappled light when awake, but do not respond when restful. **D** Sleep homeostasis in flatworms. The first 24-h (*black circles*) served as a baseline; the second 24-h (*white circles*) contained a 3-h period of forced locomotion during the day (*gray shading*), which increased activity, followed by a rebound of restfulness during the night (*black bar*). *Stars* denote significant pairwise comparisons. Reprinted with permission from Omond et al. (2017) (color figure online)

Despite the equivocal evidence for a clock in flatworms, Omond and colleagues (2017) demonstrated the presence of sleep in a freshwater flatworm (*Girardia tigrina*). When not gliding along the substrate, the animals remained motionless, contracted along their anterior–posterior axis (Fig. 2A). The flatworms were nocturnal (Fig. 2B) with dorsally placed light-detecting eye spots (or ocelli) located at the anterior end, and extraocular photoreceptors diffusely dispersed throughout their bodies (Shettigar et al. 2017, 2021) facilitating their night-active behavior. Their restful posture was thought to reflect sleep as the animals habitually avoided overhead lights when moving, yet were unresponsive when immobile (Fig. 2C). The flatworms also increased restfulness after a 3-h period of stimulation, suggestive of a sleep homeostatic response (Fig. 2D). Importantly, the increase in quiescence did not appear to arise from stress or fatigue as the amount of stimulation incurred, and the amount of movement induced, did not predict the resulting increase in restfulness (Omond et al. 2017). Taken together, these findings suggest that restful flatworms are asleep. But given all other animals studied by sleep scientists do likewise, why does it matter whether this one species of flatworm sleeps? What contribution can flatworms make to our broader understanding of sleep? To answer these questions, we must introduce other salient features of flatworm biology.

## Evolutionary relatedness and secondary simplification

**Fig. 3:**
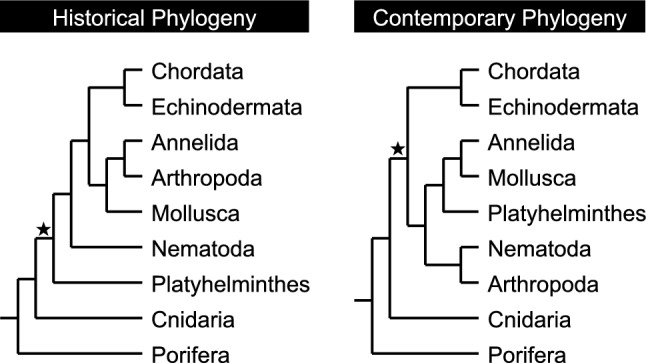
Evolutionary relationships among the 9 most species-rich animal phyla showing the historical view, based on morphology, and the modern view, based on molecular data. Flatworms were once thought to be the sister group to all other bilaterally symmetric animals, but are now considered to be a member of the derived Lophotrochozoans. As such, the simplified biology of flatworms is thought to reflect evolutionary losses. The most-recent common ancestor to all Bilaterians is marked with a *star*. Trees were adapted from Adoutte et al. (2000)

Extant flatworms were long considered to be the surviving members of the earliest branching lineage of animals exhibiting bilateral symmetry (reviewed in Adoutte et al. 2000) (Fig. 3). In the absence of genetic and genomic tools for resolving relatedness, this long-standing view on the “primitive” position of flatworms had been based on taxonomic inference. The conclusion was understandable: flatworms lack a circulatory system, respiratory system, and endocrine glands. They do not possess a coelom or an anus. Consequently, flatworms, as the sister group to all other bilaterally symmetric phyla, garnered much scientific attention as they were thought to provide insight into the evolutionary *origin* of the central (Sarnat and Netsky 1985; Halton and Gustafsson 1996; Agata et al. 1998) and peripheral (Koopowitz and Chien 1974) nervous systems, along with nerve cell types (Lentz 1967), synapse structure (Best and Noel 1969), neurochemistry (Welsh and King 1970; Welsh and Williams 1970), photoreceptors (Carpenter et al. 1974), tissue layers (Pedersen 1961), stem cells (Newmark and Sánchez Alvarado 2002), and learning (reviewed in Deochand et al. 2018).

However, the turn of the century saw molecular approaches overturn this anatomically based interpretation (Carranza et al. 1997; Adoutte et al. 2000). By doing so, Platyhelminthes were elevated from the base of the tree of Bilaterians to members of the Lophotrochozoans, a group that includes annelids and mollusks (Fig. 3); the sister group to the Lophotrochozoans is the Ecdysozoans, which includes arthropods and nematodes. This revised view of the relatedness of animals proposes that the simplified physiology of flatworms reflects — not the retention of a ‘primitive’ condition — but rather evolutionary losses through a process of secondary simplification (Paps et al. 2009).

Secondary simplification, akin to evolutionary lesions of anatomy and physiological systems, offers a compelling opportunity (O’Malley et al. 2016). What the animal retains provides insight into physiological constraints. For instance, the evolutionary appearance of flatworms arose through dorsoventral flattening of the body. Their new-found thinness permitted the diffusion of gasses across a selectively permeable body wall, supplanting the need for a coelom, and respiratory and circulatory systems. Nonetheless, flatworms retained a bilobed brain (Sarnat and Netsky 1985), sensory receptors (Shettigar et al. 2021), and a need for sleep (Omond et al. 2017).

The inability of flatworms to (evolutionarily) dispense of their need for sleep is mirrored by the retention of some—but not all—biochemical machinery that regulate sleep and wake. The neurotransmitters acetylcholine, dopamine, glutamate, and histamine promote wakefulness in vertebrates and fruit flies alike; adenosine, gamma-aminobutyric acid (GABA), and serotonin induce sleep (Stenberg 2007). The regulatory roles of these neurotransmitters appear to have been evolutionarily conserved from flies to mammals (Joiner 2016). Interestingly, while cnidarians sleep (Lesku and Ly 2017; Nath et al. 2017), sleep in the *Hydra* is regulated only by dopamine and GABA (Kanaya et al. 2020). Thus, the simplicity of cnidarians is reflected in the simplicity of their complement of neurotransmitters that regulate behavior. But do neurotransmitters in (secondarily simplified) flatworms exert the same effect as in (relatively complex) flies and mammals, with whom they share closer relatedness, or to simple *Hydra*, with whom they share more distant ancestry?

**Fig. 4:**
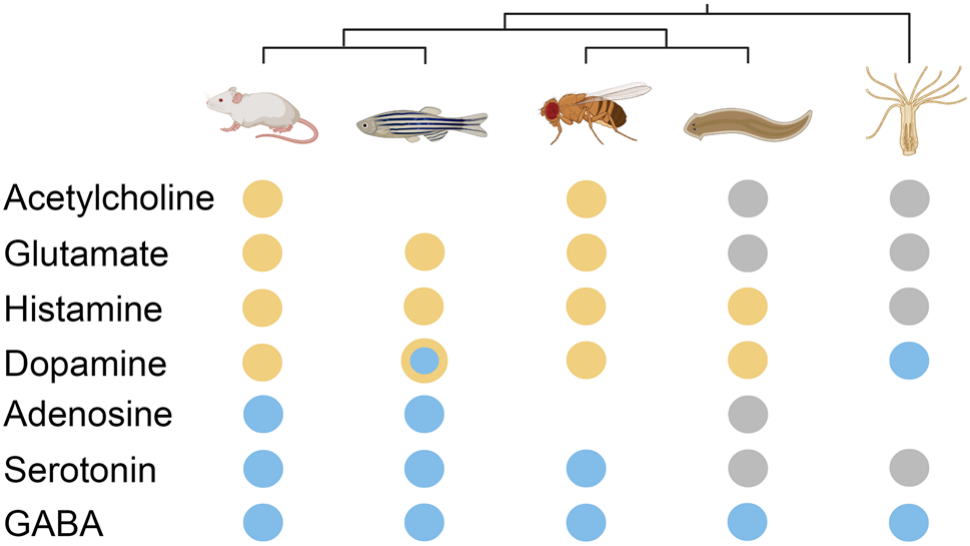
Neurotransmitters promote wakefulness (*yellow*), sleep (*blue*), or neither (*gray*) in mice, zebrafish, fruit flies, flatworms, and *Hydra*. The absence of a circle denotes an absence of data. Dopamine can promote wake or sleep in zebrafish owing to receptor-type differences. Evolutionary relationships among the five taxa are shown at the top of the illustration. Simple (*Hydra*) and secondarily simplified animals (flatworms) have fewer active neurotransmitters than more complex animals (mice, zebrafish, fruit flies). Only GABA maintains a consistent (sleep-inducing) effect across all taxa studied. Reprinted from Omond et al. (2022) (color figure online)

A recent study exposed flatworms to seven neurotransmitters, with concentrations spanning 3 to 5 orders of magnitude, and measured the amount of restfulness and distance traveled (Omond et al. 2022). Similar to *Hydra*, flatworms showed no overt response to acetylcholine, glutamate, adenosine, or serotonin (Fig. 4). Conversely, dopamine and histamine decreased restfulness and increased movement, similar to their wake-promoting effects in fruit flies and vertebrates. Consistent with an activating effect of histamine, the H1 histamine receptor antagonist, pyrilamine, induced restfulness. Lastly, GABA, whose somnogenic effect has been demonstrated in all species studied, likewise increased restfulness in flatworms in a dose-dependent manner. Accordingly, GABA inhibits neuronal activity when applied to the ventral nerve cords of the flatworm, *Notoplana acticola* (Keenan et al. 1979). GABA-mediated neuronal suppression might reflect the brain activity correlate of sleep behavior in flatworms, as sleep in other invertebrates is characterized by reduced neuronal activity (Kaiser and Steiner-Kaiser 1983; Nitz et al. 2002). Nonetheless, this prediction must be tested using respirometry (Osuma et al. 2018) and electrophysiology (Aoki et al. 2009; Freiberg et al. 2022). Notwithstanding, it appears that only GABA has held a conserved role in the regulation of sleep (Omond et al. 2022).

## Remarkable regeneration

A novel aspect of flatworm biology, with untapped potential in sleep research, is their ability to fully regenerate whole-body structures from slivers of the ‘parent’ animal (Reddien and Sánchez Alvarado 2004; Umesono and Agata 2009; Adler et al. 2014). Perhaps not surprisingly, this ability has intrigued scientists for more than a century (Morgan 1898; Hyman 1919), such that flatworms are now popular subjects in stem cell research and studies on neurodegenerative disease (Rink 2018; Ivankovic et al. 2019; Goldman and Poss 2020). Regeneration is possible because of the concerted action of stem cells called neoblasts. When injured, muscles along the wound edge contract and mucous is secreted over the wound for protection. Neoblasts then migrate to the wound edge to form a blastema, where they differentiate into the missing cell types. In this way, the animal can regenerate all body tissues, including those of the central nervous system in decapitated flatworms.

Decapitation, and subsequent regeneration, could be used to test hypotheses for sleep function. For example, the ontogenetic hypothesis proposes that sleep facilitates maturation of the developing nervous system (Roffwarg et al. 1966). This hypothesis is founded on the observation that young animals sleep more than adults (Scriba et al. 2013). If decapitated flatworms were found to sleep more than intact animals, this would provide original support for the ontogenetic hypothesis using *adult* animals. In addition to a role in neurodevelopment, sleep also saves energy by lowering the rate of oxygen consumption and through state-dependent metabolic partitioning (Schmidt 2014; Lesku and Schmidt 2022). Energy saved by sleeping can then be used for other purposes, such as reallocation to the immune system (Preston et al. 2009). Future studies could exploit the remarkable regenerative ability of flatworms to test whether flatworms become restful while regenerating to allow sleep-dependent processes to facilitate regeneration. Success in this endeavor would require a characterization of sleep based on measures beyond behavior alone, but the future is promising.

## Promising future

In addition to being a prominent behavior, sleep can also be characterized by changes in physiology. When animals sleep, metabolic rate drops, as seen in mammals (Jung et al. 2011), birds (Ferretti et al. ﻿2019), sharks (Kelly et al. 2022), and flies (Stahl et al. 2017). High-throughput respirometers for measuring oxygen consumption rates in very small invertebrates (Du Preez et al. 2020), including flatworms (Osuma et al. 2018), should allow for measurements on energy expenditure during sleep in platyhelminthes. Alternatively, owing to potential disturbance caused by the respirometer on spontaneous behavior, it might be more appropriate to induce sleep via neurotransmitters (Omond et al. 2022).

**Fig. 5:**
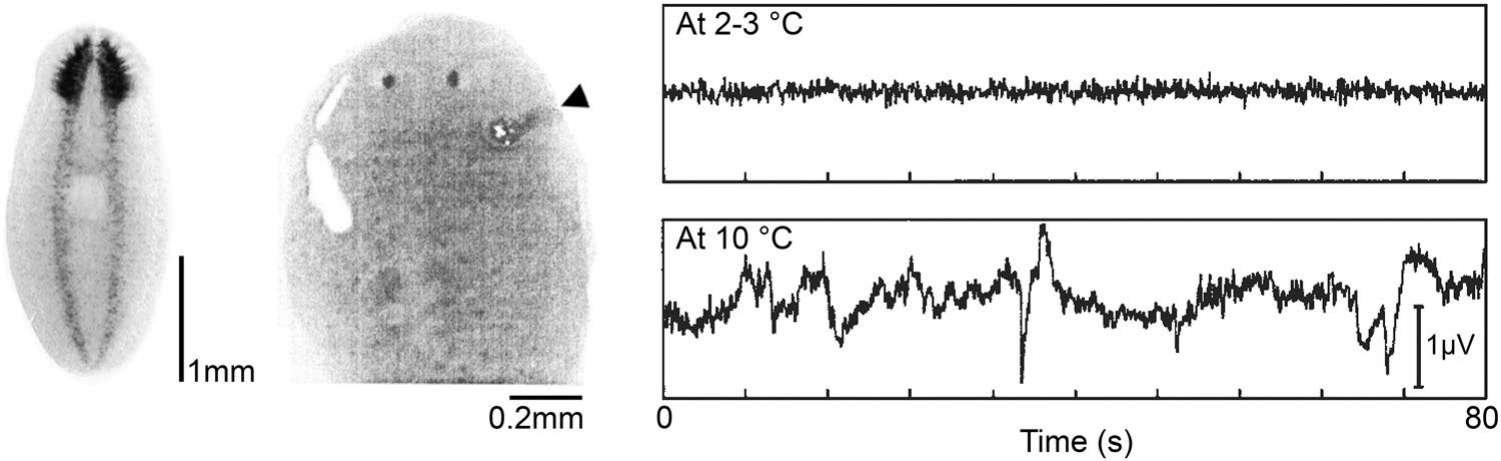
The flatworm, *Dugesia japonica*, stained with a neuron-specific probe to reveal its bilobed brain (*left*); an electrode was inserted into the brain (*arrow*) posterior to the two ocelli (*center*). Brain activity had less low-frequency activity when the animal was cold (*top right*) then after it had re-warmed; large potentials are thought to be of muscular origin (*bottom right*). Reprinted with permission from Aoki et al. (2009)

In addition to respirometry, flatworms are also amenable to recording brain activity. In birds and mammals, sleep-related changes in the electroencephalogram manifest as large, slow-waves during non-rapid eye movement (non-REM) sleep and small, wake-like, fast-waves during REM sleep (Rattenborg et al. 2022). Obtaining electrophysiological signals from the brain of sleeping invertebrates has met with surprising success (Rattenborg and Ungurean 2023). For example, optomotor interneurons in the optic lobes of honeybees (*Apis mellifera*) show lower sensitivity to moving visual stimulation during the night than during the day (Kaiser and Steiner-Kaiser 1983). Similarly, local field potentials, recorded from the medial brain in the fruit fly, reveal less activity when the flies are sleeping (Nitz et al. 2002; see also Yap et al. 2017). Brain activity has been recorded from flatworms (*Dugesia japonica*, *Schmidtea mediterranea*) using invasive (Aoki et al. 2009) and non-invasive (Freiberg et al. 2022) recording techniques. Although sleep per se was not necessarily measured in these studies, the authors were able to modulate brain activity with lights and cold temperatures (Fig. 5). Using these approaches, it should be possible to characterize the electrophysiological correlates of sleep and wakefulness in flatworms. Other approaches for visualizing in vivo brain activity, such as calcium imaging and optogenetics techniques (Turek et al. 2013; Bushey et al. 2015), are for the moment, unusable on flatworms. The chief obstacle lies in the absence of transgenic animals expressing a calcium sensor (GCaMP) (Tian et al. 2009). This hurdle may be overcome owing to the interest of geneticists in flatworm biology (Grohme et al. 2018), and more broadly, neuroscientists in non-traditional animals (Weissbourd et al. 2021).

## Conclusions

There are two motivations underlying most sleep research. One rationale seeks to identify similarities between sleep in humans and model animals. This approach pursues evolutionary homologies since any similarities may have been inherited from a common ancestor and therefore reflect physiological sameness. The other motivation explores the grand diversity of sleep through the study of any-and-all animals to describe similarities, but also *differences*, which may be salient. Here, homology has value for tracing the evolutionary history of mammalian sleep states, but this approach embraces evolutionary divergence and convergence as well. There is a need to broaden our view of sleep beyond humans, rodent strains, and fruit flies, so we may appreciate the full diversity of sleep that exists across animals (Blumberg et al. 2020; Eban-Rothschild 2022; Lesku and Rattenborg 2022; Rattenborg and Ungurean 2023).

Flatworms are one promising animal for further and extensive study. Flatworms sleep like other animals. Their sleep is regulated based on prior sleep/wake history (Omond et al. 2017) and by the action of the evolutionarily conserved neurotransmitter GABA (Omond et al. 2022). Unlike other animals, however, flatworms occupy a unique phylogenetic position, being the secondarily simplified sister taxa of more complex species (Adoutte et al. 2000). Yet, flatworms retain a centralized and peripheral nervous system, an array of sensory systems, and the ability to learn. Furthermore, they possess a remarkable ability to regenerate from a mere fragment of the ‘parent’ animal. Lastly, the use of modern, high-throughput techniques for recording metabolic rate (Osuma et al. 2018) and brain activity (Freiberg et al. 2022) highlight the flatworm for well-deserved, new-found attention in the study of sleep.
